# The Presence–Absence Situation and Its Impact on the Assemblage Structure and Interspecific Relations of Pronophilina Butterflies in the Venezuelan Andes (Lepidoptera: Nymphalidae)

**DOI:** 10.1007/s13744-012-0031-2

**Published:** 2012-05-04

**Authors:** Tomasz W Pyrcz, R Garlacz

**Affiliations:** Zoological Museum, Jagiellonian University, Kraków, Poland

**Keywords:** Altitudinal gradients, Cordillera de Merida, niche partitioning, parapatric distribution, signal jamming, spatial interference

## Abstract

Assemblage structure and altitudinal patterns of Pronophilina, a species-rich group of Andean butterflies, are compared in El Baho and Monte Zerpa, two closely situated and ecologically similar Andean localities. Their faunas differ only by the absence of *Pedaliodes ornata* Grose-Smith in El Baho. There are, however, important structural differences between the two Pronophilina assemblages. Whereas there are five co-dominant species in Monte Zerpa, including *P. ornata*, *Pedaliodes minabilis* Pyrcz is the only dominant with more than half of all the individuals in the sample in El Baho. The absence of *P. ornata* in El Baho is investigated from historical, geographic, and ecological perspectives exploring the factors responsible for its possible extinction including climate change, mass dying out of host plants, and competitive exclusion. Although competitive exclusion between *P. ornata* and *P. minabilis* is a plausible mechanism, considered that their ecological niches overlap, which suggests a limiting influence on each other’s populations, the object of competition was not identified, and the reason of the absence of *P. ornata* in El Baho could not be established. The role of spatial interference related to imperfect sexual behavioral isolation is evaluated in maintaining the parapatric altitudinal distributions of three pairs of phenotypically similar and related species of *Pedaliodes*, *Corades*, and *Lymanopoda*.

**Electronic supplementary material:**

The online version of this article (doi:10.1007/s13744-012-0031-2) contains supplementary material, which is available to authorized users.

## Introduction

In depth analysis of local butterfly assemblages structure, particularly in montane areas is often hampered by the lack of statistically workable data sets only moderately biased by sampling methods. From this perspective, the subtribe Pronophilina (Nymphalidae, Satyrinae) sensu Lamas *et al* (2004) is a particularly suitable group for studies on population ecology. It is highly diverse, constituting an overwhelming majority in terms of species richness and abundance in some high elevation areas of the northern Andes (Adams 1985). Most species are distributed in narrow elevational belts. Data on their abundance and relative frequencies are easy to obtain with standard sampling procedures (Pyrcz & Wojtusiak 2002, Pyrcz & Viloria 2007, Pyrcz *et al* 2009a, Mahecha Jimenez *et al* 2011). Pronophilina adults are easy to locate in the field as they are always associated with their larval host plants, common Andean bamboos of the genus *Chusquea* (DeVries 1987, Greeney *et al* 2009, 2010).

Synchronic occurrence of several phenotypically similar species of Pronophilina in the same habitat using the same resources suggests the possibility of competition. When the ecological niches of two, usually closely related species overlap, competitive exclusion is likely to occur leading to either the exclusive dominance of one of the two species or to coexistence (Rodríguez 2001, Juliano *et al* 2004). The theory of competitive exclusion first formulated by Gause (1934) is currently widely applied in ecology and well supported mathematically (Feng 1997, Mougi & Nishimura 2005) but rarely observed in natural ecosystems (Bengtsson 1989, Shapiro 1992). In 1996, a study of faunas and altitudinal distribution patterns of the Pronophilina was carried out in Monte Zerpa in the Venezuelan Cordillera de Mérida (Pyrcz & Wojtusiak 2002). A similar project, in scope and methods, was conducted exactly 10 years later in El Baho, a site situated in the same mountain range. The only notable difference at the species level between the two samples appeared to be the absence of one of the co-dominant species in El Baho. This finding raised two issues: How does the absence of this species affect Pronophilina assemblage structure in El Baho compared with Monte Zerpa, and what are the possible factors responsible for its absence in El Baho.

The distribution of Pronophilina species in well-defined elevational belts and the occurrence of pairs of related and/or similar species apparently replacing each other in altitude were first explored by Adams (1985) and more thoroughly investigated by Pyrcz *et al* (2009b). Adams was unable to propose an ecological mechanism responsible for maintaining altitudinal parapatric distributions. Pyrcz & Wojtusiak (2002) suggested “spatial interference” as an explanation, a mechanism somewhat related to mating interference (Feng 1997) and signal jamming (Thornhill & Alcock 1983). Mating interference and signal jamming are both interspecific interactions occurring during mate selection and that might adversely affect the fitness of one or both species involved and that is caused by incomplete species recognition (Gröning & Hochkirch 2008). The sampling in Monte Zerpa and El Baho provided an opportunity for testing this hypothesis.

## Material and Methods

### *Study area*

This study compared the cloud forest butterfly fauna of Monte Zerpa and El Baho, two sites located in the Venezuelan Cordillera de Mérida (CM), an isolated mountain range separated from the main Andes by the Táchira depression.

**Fig 1:**
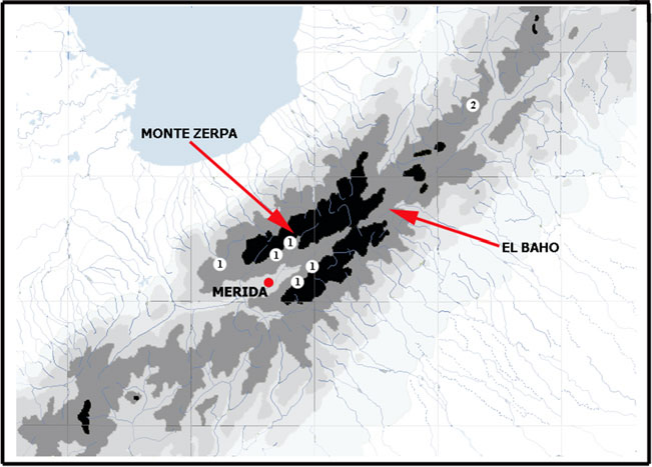
Study sites and distribution of *Pedaliodes ornata* in the Cordillera de Mérida (*1*: *Pedaliodes ornata ornata*; *2*: *Pedaliodes ornata haroldboxi*; *black* area: land above 3,000 m asl, *dark grey* area: land above 2,000 m asl).

El Baho and Monte Zerpa are 50 km apart as a crow flies. El Baho is in the upper Santo Domingo valley (8^o^50′N; 70^o^42′W), isolated from other cloud forest areas of the CM by the Serranía de Santo Domingo from the south, the Niquitao massif from the west, the Calderas massif from the north, and by the narrow and deep gorge of the Santo Domingo river from the east (Fig 1). Monte Zerpa is located in the upper Chama Valley (8^o^40′N; 71^o^8′W), separated from other cloud forests of the CM by high mountain ridges of the Serranía de la Culata from the north and the Sierra Nevada from the south, and the Ejido desert from the southwest, an area of xeric vegetation at the lower part of the valley of Chama at 700–1,300 m asl. Palynological and geological sources indicate that the Chama Valley has been isolated since the Pleistocene (Schubert & Vivas 1993). The lowest point separating the valleys of Santo Domingo and Chama is the Mucubají pass at 3,600 m asl. Cloud forests covering mid-elevations of both valleys are isolated by desert páramo above 3,200 m asl in the CM. The climate (rainfall, mean temperatures) of the two sites is similar (Veillon 1989), although daily temperature patterns show slight differences due to northwest exposure of the slope in El Baho, compared with southeast exposure of Monte Zerpa.

### *Methods*

Adult Pronophilina were sampled in El Baho along an elevational transect spanning from 2,450–3,100 m asl and extending from mid-elevation cloud forest to subparamo, a patchy habitat composed of isolated stands of tall *Swallenchloa* bamboos, Ericaceae shrubs, and scrubland (“*pajonal”*) dominated by Poaceae and Asteraceae (*Espeletia*). Butterflies were collected with standard entomological nets and in Rydon-Van Someren traps placed every 50 m in elevation. Each trap was baited with dog dung, which is a very effective attractant for adult Pronophilina butterflies (Pyrcz & Wojtusiak 2002). Each trap was checked six times in the space of 10 days. Every butterfly collected in the trap was placed in an envelope marked with the date and elevation. Sampling took place in November, which is the late rainy season in the CM. Altitude and temperature were measured every 200 m between 2,350 and 3,050 m and between the hours of 09.00–15.00 every 2 days with a Silva multifunction tool.

Data from El Baho were compared with data gathered in Monte Zerpa in 1996 during a study employing similar sampling methods. Some data from Monte Zerpa were published and discussed by Pyrcz & Wojtusiak (2002) but others remained unpublished. The Monte Zerpa transect spans 800 m in altitude, from 2,250–3,050 m, and extends from mid-elevation cloud forest to dense elfin forest dominated by *Clusia* trees and *Chusquea* bamboos.

All the individuals were identified to subspecies in the Zoological Museum of the Jagiellonian University (Kraków, Poland), which holds the largest World collection of Pronophilina, including a large-type data base containing photographs and genital dissections of types. Standard taxonomical methods were used. In particular, male and female genitalia dissections were made by soaking abdomens in warm 10% KOH solution. Genital parts were preserved in glycerol vials. Male androconia were studied through luminescent light.

**Fig 2:**
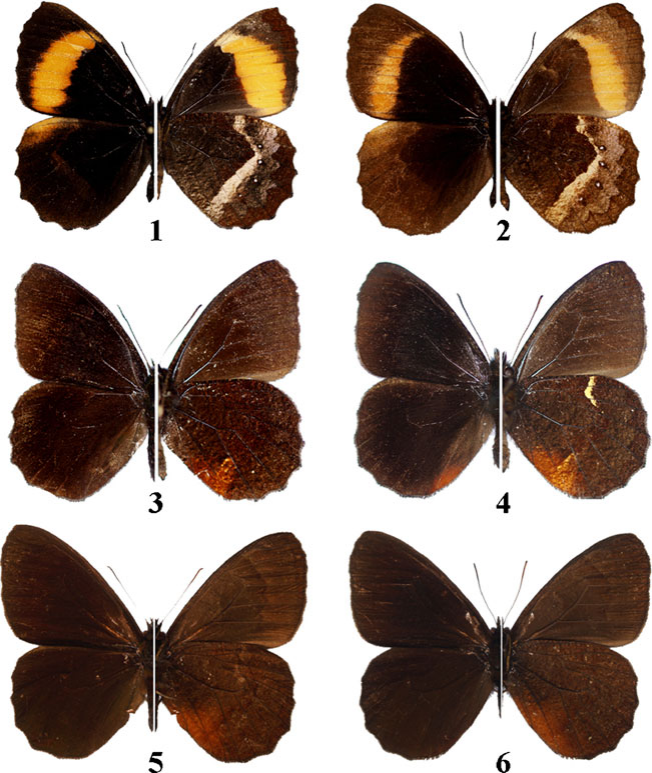
Male adults of *Pedaliodes ornata*, *Pedaliodes minabilis*, and *Pedaliodes montagna. 1*. *Pedaliodes ornata haroldboxi*, Pico Tonojo, *2*. *Pedaliodes ornata ornata*, Monte Zerpa, *3*. *Pedaliodes minabilis*, Monte Zerpa, *4*. *Pedaliodes minabilis*, El Baho (form *luteocosta*), *5*. *Pedaliodes montagna*, Monte Zerpa, *6*. *Pedaliodes montagna*, El Baho.

Distributional (altitudinal and geographic) patterns of all species reported from El Baho and Monte Zerpa were examined based on data gathered throughout the CM by the senior author between 1992 and 2010. Some of the distributional data were published in a series of taxonomical and zoogeographical papers (Viloria *et al* 2003, Pyrcz 2007, Pyrcz & Viloria 2009, Pyrcz *et al* 2009a, 2010), whereas others remain unpublished. A special attention was given to three species of *Pedaliodes*: *P. ornata* Grose-Smith, *P. minabilis* Pyrcz, and *Pedaliodes montagna* Adams & Bernard (Fig 2). *Pedaliodes ornata* is an endemic species in the CM with a disjunctive distribution (Fig 1). It was recorded in two areas. The nominate subspecies occurs in the valley of Chama in the central part of the range and on the Lago de Maracaibo slopes of the La Culata. *Pedaliodes ornata haroldboxi* Pyrcz & Viloria is found only in the Pico Tonojo massif in the northern CM. *Pedaliodes minabilis*, previously confused with *P. ferratilis* Butler (Adams & Bernard 1981), is also an endemic species of the CM. It is found throughout the range, including the Chama and Santo Domingo valleys. The Chama valley population is characterized by a uniform HWV color pattern. The other form, *Pedaliodes minabilis* f. *luteocosta*, has a yellow HWV costal streak and is rare in the Chama valley but predominates elsewhere (all but two specimens in El Baho). *Pedaliodes montagna*, described originally from the CM, has since been found to be widely distributed in the Andes (Pyrcz 2004). *Pedaliodes montagna* and *P. minabilis* are similar in size and color patterns; they are both predominantly dark-brown-patterned on the upper and underside. Sexual dimorphism in *P. minabilis*, *P. montagna*, and *P. ornata* is slight and expressed only in the slightly larger size of the females.

A list of all the species recorded in El Baho and Monte Zerpa was compiled. The Sørensen index was used to assess similarity at the species and subspecies level. A statistical analysis of the data set composed of trapped individuals was performed. Shannon (H’) and Simpson (D) diversity measures, Berger–Parker index of dominance and evenness measure, and Fisher’s Alpha index of diversity were calculated for the two transects. Proportions of *P. minabilis* and *P. ornata* in the samples were calculated. Distribution patterns along altitude for *P. minabilis* and *P. montagna, Lymanopoda obsoleta* Westwood and *Lymanopoda dietzi* Adams & Bernard, and *Corades chelonis* Hewitson and *Corades pax* Watkins were measured. Spearman correlation (*S*_corr_) was computed to correlate altitudinal distribution patterns of the above species with each other. Relative abundance of *P. minabilis* and *P. ornata* were calculated. Non-parametric species-richness estimators were considered uninformative and were not used because the samples represent complete faunal inventories (an assumption based on the knowledge of distributional patterns of Pronophilina in the CM). Statistical analysis was carried out with the Statistica 6.0 software package (Tulsa, USA) and Species Diversity and Richness III software (Pisces Conservation).

## Results

### *Fauna*

A total of 22 species were collected in baited traps along the elevational transect in El Baho (see Online supplementary material). Additionally, four species, *Lymanopoda albocincta, Pedaliodes proerna, Pedaliodes japhleta* Butler, and *Corades pannonia* Hewitson were collected exclusively in hand nets at 2,300–2,400 m and one, *Manerebia franciscae* (Adams & Bernard), at 2,550 m asl. In Monte Zerpa, 22 species were also collected on baits along the transect (see Online supplementary material). Three species, *Lymanopoda marianna* Staudinger*, Steromapedaliodes albonotata* (Godman), and *Cheimas opalinus* (Staudinger), were caught in hand nets at 3,100 m asl. The only species reported in Monte Zerpa and not found in El Baho was *P. ornata*. Its absence is not an artifact of the sampling method used, as the elevation belt where it occurs (2,400–2,900 m asl) was extensively sampled in El Baho with both baited traps and hand nets before and after this study during different seasons of the year. Sørensen similarity index between El Baho and Monte Zerpa is 0.74 at the species level for trapped species and 0.96 when species collected with hand nets were included. At the subspecies level, the Sørensen similarity index is 0.55. The latter lower value reflects a wide scale differentiation of the Pronophilina of the CM at high elevations (above 2,800 m asl), which has led to the evolution of several sub-centers of endemism throughout the range. Six species, including four paramo and subparamo specialists, were represented in El Baho and Monte Zerpa by different subspecies: *C. opalinus, L. marianna*, *S*. *albonotata, L. dietzi, Lasiophila zapatoza* (Westwood), and *Pedaliodes plotina* (Hewitson).

### *Diversity and assemblage structure*

In El Baho, 543 individuals were collected in baited traps over six sampling days (ca. 90 per day) compared with 943 individuals in Monte Zerpa over 20 days (ca. 47 per day). Sampling took place in the dry season in Monte Zerpa and in the wet season in El Baho. Although Pronophilina phenology has not been rigorously studied so far, our experience in the field indicates that abundance is higher during dry season, but there are few differences in seasonal species composition and species relative abundance. Most seasonal species belong to the genus *Manerebia* and to paramo denizens. Diversity in both Monte Zerpa and El Baho is low. Particularly in El Baho, the Shannon index indicates an overall value below 2 and along the transect values below 2 at 13 from among 15 elevational stations except for the lowest two at 2,400 and 2,450 m asl (Table 1). There is a negative correlation between species richness and elevation in El Baho (*S*_corr_ = −0.54, *P* < 0.003), but there is no correlation between diversity (H) and elevation (*S*_corr_ = 0.26, *P* > 0.34). In Monte Zerpa, the Shannon diversity index indicates slightly higher values at all elevational stations. The overall Shannon index is higher in Monte Zerpa, but the Fisher Alpha and Simpson indices are lower (Table 2). The Berger–Parker dominance index is very low in both sites but particularly so in El Baho. In both Monte Zerpa and El Baho, species abundance agrees with log series model (EB, *P* = 0.13; MZ, *P* = 0.07), which justifies the use of Fisher Alpha as the most appropriate measure of diversity. Moreover, the “broken stick model” fitted only for Monte Zerpa sample (*P* = 0.81) showing structural difference in comparison with El Baho sample (*P* = 0.01) and agrees with the observed higher evenness in Monte Zerpa.

**Table 1:** Species richness (S), Shannon diversity (H’) indices along elevational transects in El Baho (EB) and Monte Zerpa (MZ).

		2250 m	2300 m	2350 m	2400 m	2450 m	2500 m	2550 m	2600 m	2650 m	2700 m	2750 m	2800 m	2850 m	2900 m	2950 m	3000 m	3050 m	3100 m
S	EB	–	–	–	10	16	10	7	5	5	6	4	4	6	5	6	6	1	6
	MZ	3	11	11	13	13	13	13	14	10	11	9	7	6	6	4	5	4	–
H	EB	–	–	–	2.21	2.34	1.38	0.87	1.08	1.13	1.71	0.45	0.46	0.89	1.52	1.49	1.67	0.00	1.45
	MZ	1.01	2.06	2.2	2.18	2.13	2.14	1.98	2.14	2.11	2.08	1.81	1.63	1.57	1.35	0.83	1.31	1.15	–

**Table 2:** Overall diversity indices in Monte Zerpa and El Baho.

	Fisher alpha	Shannon	Berger–Parker	Simpson	Evenness
Monte Zerpa	3.52	2.45	0.16	0.11	0.57
El Baho	4.6	1.88	0.02	0.31	0.42

At the assemblage level, the most notable differences are the dominance of *P. minabilis* in the El Baho sample (53 % overall and >60 % of the sample at seven elevations) and the absence of *P. ornata* in El Baho, one of the co-dominant species in Monte Zerpa (Table 3). In Monte Zerpa, *P. minabilis* + *P. ornata* are co-dominant species at 2,800–3,000 m asl (Table 4). In El Baho, the second most abundant species is *C. pax* (10.4 %), the only other one with more than 10 % in the sample. The second most abundant species of *Pedaliodes* in El Baho is *P. montagna* represented by 7 %. In Monte Zerpa, *P. montagna* (17 %) is the most abundant species (Table 5). It is the dominant species in the *Pedaliodes* community at lower elevations (below 2,500 m). *Pedaliodes montagna* also occurs at lower elevations (below 2,600 m asl) in El Baho. *Pedaliodes minabilis* is the third most abundant species in Monte Zerpa.

**Table 3:** Percentages of species in the samples in Monte Zerpa and El Baho (collected in baited traps along altitudinal transects), species with more than 10 % in the sample are highlighted in bold.

	El Baho	Monte Zerpa
*Cheimas* *opalinus*	1.8 %	–^a^
*Corades* *chelonis*	1.3 %	5.3 %
*Corades* sp.	0.9 %	–^a^
*Corades medeba*	0.6 %	0.5 %
*Corades panonia*	–^c^	0.9 %
***Corades pax***	**11**.**4 %**	2.4 %
***Eretris porphyria***	1.5 %	**15**.**5 %**
*Lasiophila* *zapatoza*	0.7 %	2.8 %
*Lymanopoda* *albocincta*	–^c^	1.0 %
*Lymanopoda* *dietzi*	3.5 %	3.4 %
*Lymanopoda* *marianna*	0.6 %	–^a^
*Lymanopoda* *obsoleta*	0.4 %	4.2 %
*Manerebia* *franciscae*	0.6 %	–^e^
*Mygona irmina*	1.1 %	1.7 %
*Pedaliodes fumaria*	–^c^	0.1 %
*Pedaliodes* *japhleta*	0.4 %	6.2 %
*Pedaliodes jephtha*	–^d^	0.1 %
***Pedaliodes*** ***minabilis***	**53**.**4 %**	**14**.**1 %**
***Pedaliodes*** ***montagna***	7.0 %	**17**.**0 %**
***Pedaliodes*** ***ornata***	**–**	**10**.**4 %**
*Pedaliodes panyasis*	2.0 %	0.5 %
*Pedaliodes* *plotina*	2.8 %	–^b^
*Pedaliodes polla*	1.8 %	0.3 %
*Pronophila epidipnis*	4.4 %	3.2 %
*Pronophila unifasciata*	0.2 %	–^b^
***Steroma bega***	2.2 %	**10**.**3 %**
*Steromapedaliodes* *albonotata*	1.5 %	–^a^

^a^Collected in hand net above 3,050 m asl.

^b^Collected in hand net below 2,250 m asl.

^c^Collected in hand net below 2,400 m asl.

^d^Collected in hand net at 2,550 m asl.

^e^Collected in hand net at 2,600 m asl.

**Table 4:** Proportions of *Pedaliodes minabilis* and *Pedaliodes ornata* in elevational transects samples.

	2400 m	2450 m	2500 m	2550 m	2600 m	2650 m	2700 m	2750 m	2800 m	2850 m	2900 m	2950 m	3000 m	3050 m	3100 m
*Pedaliodes minabilis* Monte Zerpa	0	0	0	0.02	0.09	0.12	0.05	0.11	0.31	0.43	0.54	0.75	0.5	0.57	–
*Pedaliodes minabilis + Pedaliodes ornata* Monte Zerpa	0	0	0	0.03	0.14	0.24	0.26	0.38	0.61	0.59	0.73	0.82	0.58	0.71	–
*Pedaliodes minabilis* El Baho	0.14	0.21	0.63	0.78	0.62	0.60	0.2	0.89	0.89	0.76	0.22	0.43	0.12	0.47	0.47

**Table 5:** Number of species along elevational transects per classes of abundances.

	0.1–3 %	3.1–6 %	6.1–12 %	12.1–24 %	24.1–48 %	>48 %
El Baho	17	2	2	0	0	1
Monte Zerpa	10	4	3	3	0	0

### *Altitudinal patterns*

**Fig 3:**
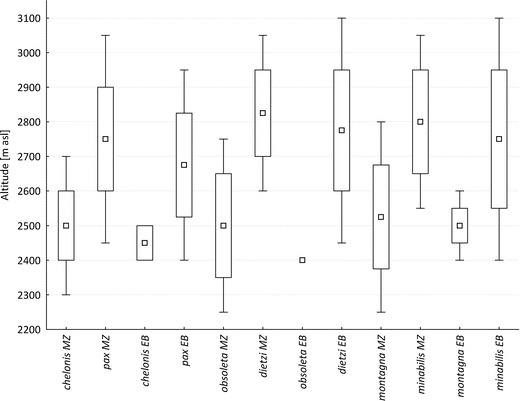
Altitudinal distribution patterns of three pairs of parapatric species in Monte Zerpa (*MZ*) and El Baho (*EB*). *Box* and *whiskers* plot with median, lower, and upper quartile, minimum and maximum and outside values of altitude range.

In El Baho, several species occur some 150–200 m lower than in Monte Zerpa, including *P. minabilis* and *L. dietzi*, and the high-elevation species not recorded in traps in Monte Zerpa below 3,050 m asl, *C. opalinus, L. marianna,* and *S. albonotata*. Three pairs of species (*P. minabilis* and *P. montagna, L. obsoleta* and *L. dietzi*, and *C. chelonis* and *C. pax*) recorded in Monte Zerpa and replacing each other in elevation are also found in El Baho. *Lymanopoda dietzi* and *L. obsoleta* overlap at 2,600–2,750 in Monte Zerpa and come into contact at 2,400–2,450 m asl in El Baho. *Corades pax* and *C. chelonis* overlap in Monte Zerpa at 2,450–2,700 m asl and at 2,400–2,500 m asl in El Baho. *Pedaliodes minabilis* and *P. montagna* overlap at 2,550–2,800 in Monte Zerpa and 2,400–2,600 in El Baho. Altitudinal ranges of *P*. *minabilis* and *P. ornata* in Monte Zerpa overlap and show a strong positive correlation (*S*_corr_ = 0.86; *P* < 0.001) (Fig 3).

## Discussion

Species composition in Monte Zerpa and El Baho is almost identical, which agrees with predictions based on geographical proximity of the two sites and the knowledge of distribution patterns of Pronophilina in the CM. The only difference between the faunas is the absence of *P*. *ornata* in El Baho. *Pedaliodes ornata* is an important absentee in El Baho considering that it is one of five co-dominant species in the closely situated Monte Zerpa. Consequently, the assemblages of Pronophilina in El Baho and Monte Zerpa are considerably different structurally and also in the altitudinal distribution patterns of individuals species.

Structurally, the sample in El Baho is unlike other studies of Pronophilina in that one species, *P. minabilis*, accounts for more than half of all specimens. The sample in Monte Zerpa is much more typical with no single species domination and five co-dominant species representing together over 60 % of all specimens (Pyrcz & Wojtusiak 1999, 2002, Pyrcz 2004, Pyrcz *et al* 2009b, Mahecha Jimenez *et al* 2011). The models of species abundance show best fit for broken stick model for Monte Zerpa, which agrees with the assumption that it describes the assemblages with no single species dominance and high evenness. The low evenness in El Baho and the overwhelming dominance of one species could be interpreted as the sign of disturbed ecosystem.

It is unlikely that *P. ornata* has never occurred in El Baho because of its present-day distribution south and north of the Santo Domingo valley and because it is the only species of nearly 40 species of Pronophilina in the CM with such a disjunct distribution. The hypothesis that *P. ornata* went extinct in El Baho could be due to at least four major extrinsic factors: habitat loss, mass dying-off of host plants, selective pressure, or competitive exclusion. None of these can be tested at present, but their plausibility can be evaluated. Climate shift, such as dry or/and colder periods in the Pleistocene and Holocene widely documented for the CM (Schubert & Vivas 1993), may cause local disappearance of cloud forests and cause changes in abundance or even lead to the extinction of species. It is also known that mass blooming and simultaneous dying out of *Chusquea* (Poaceae) bamboos over large portions of land, occurring every 10–40 years (Judziewicz *et al* 1999), may affect the abundance and distribution of Pronophilina (DeVries 1987). We observed such a dying out on several occasions in the Ecuadorian, Peruvian, and Venezuelan Andes (unpublished). Both phenomena act, however, non-selectively on all the species associated within a given habitat. They would therefore almost certainly have driven the extinction of all or at least some of the species occurring in the same elevational band as *P. ornata* in El Baho. This, however, is not the case, as all other eight species found within this band in El Baho are distributed throughout the CM. Therefore, climate shift and bamboo die out events can be reasonably ruled out as factors responsible for the extinction of *P. ornata* in El Baho. The third factor implies different selective pressures involving species-specific agents, predators, or parasitoids, in El Baho and Monte Zerpa. Given our current knowledge, this seems unlikely considering the geographic proximity and similar ecological conditions of the two sites. Species-specific predation by birds could play a role, especially if the two different phenotypes coexist at sharply different frequency (Allen & Anderson 1984, Lindström *et al* 2001). It does not explain, however, how the frequency ratio would shift in favor of *P. minabilis* in El Baho from the balanced co-domination in Monte Zerpa. External parasites, such as ticks of the family Erythraeidae, are commonly found attached to *Pedaliodes* bodies and wings, but they are not known to be species-specific (Gabryś 2000 and personal communication).

A fourth factor could be interspecific competition and the elimination of one species by the other. The idea of competitive exclusion involves two fundamental issues: delimitation of the niche and identification of the item of competition. Ecological niche is the activity range of each species along every dimension of the environment (Hutchinson 1957). *Pedaliodes ornata* and *P. minabilis* have closely similar altitudinal distributions which are considered as important dimensions of Pronophilina ecological niches (Pyrcz & Wojtusiak 2002). This could be interpreted as indirect evidence that the two species limit each other. Thus, some kind of spatial partitioning/competition certainly takes place. Indeed, the absence of *P. ornata* in El Baho appears to be advantageous for *P. minabilis* because its ecological niche is wider, as shown by higher abundance and wider elevational span. The competition item is most commonly defined as the “limited resource”, often food sources (Peck & Howden 1984, Ciroz-Pérez *et al* 2002, Juliano *et al* 2004). For example, dung is considered a limited resource in cases of competitive exclusion among adult Scarabaeidae beetles distributed along an elevational gradient (Hanski & Niemela 1990, Escobar *et al* 2005). Host plants may also be a limited resource for the larvae of phytophagous insects (Denno *et al* 1995, Reis *et al* 1999), especially host specialized monophages. Interspecific food competition among the (mostly oligophagous) larvae of *Pedaliodes* feeding on common cloud forests plants, which often dominate plant communities, has not been recorded, however. Despite a wealth of ecological studies focusing on diurnal Lepidoptera, there is little evidence for resource competition in adult butterflies, except for mating and courtship sites, especially among males that use perches, light gaps, or clearings to enhance their mating chances (Davies 1978, Thornhill & Alcock 1983). However, competition for space between males of *Pedaliodes* was not observed (Pyrcz 2004). It is worth to point out that there are few species of *Pedaliodes*, up to four, in the uppermost cloud forests in the CM. Elsewhere in the Andes, up to 20 species coexist within the same elevational band, meaning more complex interspecific interactions, and, perhaps, also a greater carrying capacity in such region (Pyrcz & Wojtusiak 1999, Pyrcz 2004). Interspecific competition increases with increasing phenotypic similarity and finally equals infraspecific competition, which is the highest value of the competition coefficient (Rodríguez 2001). Markedly different color patterns contribute to the phenotypic dissimilarity of *P. ornata* and *P. minabilis*, consequently to their lower competition value. In the light of the above, competitive exclusion between two species of *Pedaliodes* seen from the traditional perspective is unlikely and remains a theoretical challenge. Notwithstanding, possible extinction of *P. ornata* in El Baho could have an important role in shaping the local Pronophilina assemblage structure by breaking a balanced two species co-domination at higher elevations and enabling *P. minabilis* to occupy its ecological niche.

Pyrcz & Wojtusiak (2002) pointed out that in Monte Zerpa three pairs of closely related species have mutually exclusive distributions: *P*. *montagna* and *P. minabilis*, *L*. *dietzi* and *L. obsoleta*, and *Corades chelonis* and *C. pax*. All of them also occur in El Baho and replace each other in elevation, although, statistically, this cannot be demonstrated because the sample is not numerous enough. Signal jamming theory rests on the fact that most butterflies, and certainly those belonging to the subfamily Satyrinae (Braby & New 1988), first locate their mates visually and then use contact pheromones once courtship has ensued (Rutowski 1997).

If two species are phenotypically similar (colors, size, flight pattern, etc.) and have overlapping ranges, individuals of one species may falsely identify the other species as a potential mate. This leads to fruitless interspecific interactions involving pursuit, courtship, and consequently to time and energy loss. Such interactions can be frequently observed in nature. In the overlapping zone, the chances of meeting an individual of its own species (*p*) can be expressed by the simple equation: $p = a1/a2 + a{1}$, where *a*1 and *a*2 are abundances of parapatric species. At the abundance equilibrium, the point where two species occur at equal numbers, *p* = 1/2. The *p* coefficient decreases with the distance from the equilibrium point, and interspecific interactions resulting in time and energy loss are more frequent, whereas the chances of meeting a conspecific, and successful mating are fewer. Hence, individual fitness is lower. Thus, the occurrence of individuals of one species is a buffer for the spreading of non-conspecifics and extending their species range. The two species exert on each other a dynamic, demographic pressure. Altitudinal distribution patterns may shift when the balance of abundance is disturbed, for example, by the extinction of species or the dying-out of host plants in a given elevational band. In fact, in all three cases of parapatric species their overlapping zone in El Baho is situated some 200–250 m lower than in Monte Zerpa. We assume that this mechanism may occur in case of parapatric species, whose behavioral sexual isolation mechanisms are imperfect, rather than among sympatric species with widely overlapping ranges. Pyrcz & Wojtusiak (2002) put out a case strongly suggesting that closely related parapatric species have an effect on the width of the elevational range of each other. *Corades chelonis* occurs up to 2,500–2,700 m asl throughout the CM and is replaced at higher elevations by *C. pax*. In the neighboring El Tamá massif, where neither *C. pax* nor any other closely related species exists, *C. chelonis* occurs to timberline at 3,000–3,100 m asl. In the same range, *L. obsoleta* occurs up to 2,400–2,600 m asl and is replaced at higher elevations by its sister species *Lymanopoda lecromi* Pyrcz & Viloria (Pyrcz & Viloria 2007, Casner & Pyrcz 2010, Pyrcz *et al* 2010). However, in the Bolivian Yungas, where *L. obsoleta* has no upper parapatric ally, it occurs at least up to 3,000 m asl, therefore near timberline. In northern Peru, *Corades cistene* Hewitson occurs to at least 3,200 m asl (Pyrcz 2004), whereas in south-east Ecuador, where its range overlaps with that of its sister species *Corades dymantis* Thieme, it occurs only up to 2,400–2,500 m asl and is replaced at higher elevations by the latter species (Pyrcz 2010). Spatial interference is a mechanism which may apply to many other cases of closely related species of butterflies distributed parapatrically in adjacent altitudinal bands and indeed to other taxa (Shapiro 1992). This hypothesis needs to be tested, although securing direct experimental evidence bearing on the problem will be exceedingly difficult.

## Electronic supplementary material

Below is the link to the electronic supplementary material.

ESM 1 (DOC 104 kb)
