# Supplementary material for: The Presence–Absence Situation and Its Impact on the Assemblage Structure and Interspecific Relations of Pronophilina Butterflies in the Venezuelan Andes (Lepidoptera: Nymphalidae)
Source: Neotrop Entomol. 2012 May 4;41(3):186–95. doi: 10.1007/s13744-012-0031-2 (PMC3380249; doi:10.1007/s13744-012-0031-2)
Supplement: Supplementary file 1 — (DOC 104 kb) [file 13744_2012_31_MOESM1_ESM.doc]

Online Supplementary Materail

Data matrix

|  | *P.fumaria* | *C.chelonis* | *L.zapatoza* | *L.albocincta* | *S.bega* | *L.dietzi* | *P.epidipnis* | *P.minabilis* | *M.irmina* | *P.japhleta* | *P.jephtha* | *C.medeba* | *P.montagna* | *L.obsoleta* | *P.ornata* | *C.pannonia* | *P.panyasis* | *C.pax* | *P.polla* | *E.porphyria* | *Ch.opalinus* | *S.albonotata* | *C.sp.* | *L.marianna* | *M.franciscae* | *P.plotina* | *P.unifasciata* |
| --- | --- | --- | --- | --- | --- | --- | --- | --- | --- | --- | --- | --- | --- | --- | --- | --- | --- | --- | --- | --- | --- | --- | --- | --- | --- | --- | --- |
|  | El Baho | | | | | | | | | | | | | | | | | | | | | | | | | | |
| 2400 | 0 | 1 | 1 | 0 | 0 | 0 | 1 | 2 | 0 | 0 | 1 | 0 | 3 | 2 | 0 | 0 | 1 | 1 | 1 | 0 | 0 | 0 | 0 | 0 | 0 | 0 | 0 |
| 2450 | 0 | 5 | 3 | 0 | 4 | 1 | 13 | 27 | 6 | 0 | 1 | 1 | 23 | 0 | 0 | 0 | 4 | 18 | 4 | 3 | 0 | 0 | 0 | 0 | 3 | 12 | 0 |
| 2500 | 0 | 1 | 0 | 0 | 1 | 0 | 5 | 48 | 0 | 0 | 0 | 1 | 7 | 0 | 0 | 0 | 3 | 6 | 0 | 2 | 0 | 0 | 0 | 0 | 0 | 2 | 0 |
| 2550 | 0 | 0 | 0 | 0 | 0 | 0 | 2 | 55 | 0 | 0 | 0 | 0 | 3 | 0 | 0 | 0 | 3 | 5 | 1 | 0 | 0 | 0 | 0 | 0 | 0 | 0 | 1 |
| 2600 | 0 | 0 | 0 | 0 | 0 | 2 | 0 | 20 | 0 | 0 | 0 | 0 | 2 | 0 | 0 | 0 | 0 | 7 | 0 | 0 | 0 | 0 | 0 | 0 | 0 | 1 | 0 |
| 2650 | 0 | 0 | 0 | 0 | 2 | 3 | 1 | 20 | 0 | 0 | 0 | 0 | 0 | 0 | 0 | 0 | 0 | 7 | 0 | 0 | 0 | 0 | 0 | 0 | 0 | 0 | 0 |
| 2700 | 0 | 0 | 0 | 0 | 3 | 5 | 1 | 4 | 0 | 0 | 0 | 0 | 0 | 0 | 0 | 0 | 0 | 4 | 0 | 3 | 0 | 0 | 0 | 0 | 0 | 0 | 0 |
| 2750 | 0 | 0 | 0 | 0 | 0 | 0 | 1 | 26 | 0 | 0 | 0 | 1 | 0 | 0 | 0 | 0 | 0 | 1 | 0 | 0 | 0 | 0 | 0 | 0 | 0 | 0 | 0 |
| 2800 | 0 | 0 | 0 | 0 | 0 | 1 | 0 | 25 | 0 | 0 | 0 | 0 | 0 | 0 | 0 | 0 | 0 | 1 | 0 | 0 | 1 | 0 | 0 | 0 | 0 | 0 | 0 |
| 2850 | 0 | 0 | 0 | 0 | 2 | 3 | 0 | 45 | 0 | 0 | 0 | 0 | 0 | 0 | 0 | 0 | 0 | 6 | 1 | 0 | 2 | 0 | 0 | 0 | 0 | 0 | 0 |
| 2900 | 0 | 0 | 0 | 0 | 0 | 0 | 0 | 2 | 0 | 0 | 0 | 0 | 0 | 0 | 0 | 0 | 0 | 2 | 1 | 0 | 3 | 0 | 1 | 0 | 0 | 0 | 0 |
| 2950 | 0 | 0 | 0 | 0 | 0 | 2 | 0 | 7 | 0 | 0 | 0 | 0 | 0 | 0 | 0 | 0 | 0 | 4 | 0 | 0 | 1 | 0 | 1 | 1 | 0 | 0 | 0 |
| 3000 | 0 | 0 | 0 | 0 | 0 | 1 | 0 | 1 | 0 | 0 | 0 | 0 | 0 | 0 | 0 | 0 | 0 | 0 | 1 | 0 | 1 | 0 | 3 | 1 | 0 | 0 | 0 |
| 3050 | 0 | 0 | 0 | 0 | 0 | 0 | 0 | 0 | 0 | 0 | 0 | 0 | 0 | 0 | 0 | 0 | 0 | 0 | 0 | 0 | 0 | 4 | 0 | 0 | 0 | 0 | 0 |
| 3100 | 0 | 0 | 0 | 0 | 0 | 1 | 0 | 8 | 0 | 0 | 0 | 0 | 0 | 0 | 0 | 0 | 0 | 0 | 1 | 0 | 2 | 4 | 0 | 1 | 0 | 0 | 0 |
|  | Monte Zerpa | | | | | | | | | | | | | | | | | | | | | | | | | | |
| 2250 | 0 | 0 | 0 | 0 | 0 | 0 | 0 | 0 | 0 | 1 | 0 | 0 | 2 | 3 | 0 | 0 | 0 | 0 | 0 | 0 | 0 | 0 | 0 | 0 | 0 | 0 | 0 |
| 2300 | 0 | 2 | 4 | 1 | 2 | 0 | 3 | 0 | 5 | 6 | 0 | 1 | 5 | 2 | 0 | 0 | 0 | 0 | 0 | 16 | 0 | 0 | 0 | 0 | 0 | 0 | 0 |
| 2350 | 0 | 4 | 1 | 4 | 0 | 0 | 1 | 0 | 2 | 4 | 0 | 1 | 3 | 7 | 0 | 1 | 0 | 0 | 0 | 3 | 0 | 0 | 0 | 0 | 0 | 0 | 0 |
| 2400 | 1 | 7 | 6 | 0 | 4 | 0 | 1 | 0 | 5 | 16 | 0 | 0 | 18 | 5 | 0 | 1 | 1 | 0 | 0 | 7 | 0 | 0 | 0 | 0 | 0 | 0 | 0 |
| 2450 | 0 | 11 | 2 | 2 | 3 | 0 | 5 | 0 | 4 | 8 | 0 | 0 | 25 | 4 | 0 | 3 | 0 | 1 | 0 | 16 | 0 | 0 | 0 | 0 | 0 | 0 | 0 |
| 2500 | 0 | 10 | 5 | 2 | 6 | 0 | 7 | 0 | 1 | 12 | 0 | 1 | 21 | 4 | 0 | 1 | 2 | 0 | 0 | 22 | 0 | 0 | 0 | 0 | 0 | 0 | 0 |
| 2550 | 0 | 8 | 1 | 1 | 16 | 0 | 12 | 2 | 0 | 5 | 1 | 0 | 33 | 2 | 1 | 2 | 0 | 0 | 0 | 14 | 0 | 0 | 0 | 0 | 0 | 0 | 0 |
| 2600 | 0 | 7 | 2 | 0 | 14 | 1 | 4 | 10 | 0 | 8 | 0 | 0 | 38 | 4 | 6 | 1 | 1 | 4 | 0 | 7 | 0 | 0 | 0 | 0 | 0 | 0 | 0 |
| 2650 | 0 | 4 | 4 | 0 | 14 | 3 | 0 | 11 | 0 | 4 | 0 | 0 | 2 | 4 | 11 | 0 | 0 | 0 | 0 | 14 | 0 | 0 | 0 | 0 | 0 | 0 | 0 |
| 2700 | 0 | 1 | 4 | 0 | 16 | 5 | 0 | 4 | 0 | 0 | 0 | 0 | 4 | 7 | 16 | 0 | 0 | 3 | 1 | 15 | 0 | 0 | 0 | 0 | 0 | 0 | 0 |
| 2750 | 0 | 0 | 0 | 0 | 5 | 3 | 0 | 6 | 0 | 0 | 0 | 0 | 4 | 1 | 15 | 0 | 0 | 1 | 2 | 17 | 0 | 0 | 0 | 0 | 0 | 0 | 0 |
| 2800 | 0 | 0 | 0 | 0 | 10 | 14 | 0 | 38 | 0 | 0 | 0 | 0 | 1 | 0 | 38 | 0 | 0 | 6 | 0 | 17 | 0 | 0 | 0 | 0 | 0 | 0 | 0 |
| 2850 | 0 | 0 | 0 | 0 | 10 | 6 | 0 | 29 | 0 | 0 | 0 | 0 | 0 | 0 | 11 | 0 | 0 | 5 | 0 | 6 | 0 | 0 | 0 | 0 | 0 | 0 | 0 |
| 2900 | 0 | 0 | 0 | 0 | 3 | 2 | 0 | 14 | 0 | 0 | 0 | 0 | 0 | 0 | 5 | 0 | 0 | 1 | 0 | 1 | 0 | 0 | 0 | 0 | 0 | 0 | 0 |
| 2950 | 0 | 0 | 0 | 0 | 2 | 0 | 0 | 21 | 0 | 0 | 0 | 0 | 0 | 0 | 2 | 0 | 0 | 0 | 0 | 3 | 0 | 0 | 0 | 0 | 0 | 0 | 0 |
| 3000 | 0 | 0 | 0 | 0 | 1 | 0 | 0 | 6 | 0 | 0 | 0 | 0 | 0 | 0 | 1 | 0 | 0 | 3 | 0 | 1 | 0 | 0 | 0 | 0 | 0 | 0 | 0 |
| 3050 | 0 | 0 | 0 | 0 | 0 | 1 | 0 | 4 | 0 | 0 | 0 | 0 | 0 | 0 | 1 | 0 | 0 | 1 | 0 | 0 | 0 | 0 | 0 | 0 | 0 | 0 | 0 |
